# Performance of SARS COV-2 IgG Anti-N as an Independent Marker of Exposure to SARS COV-2 in an Unvaccinated West African Population

**DOI:** 10.4269/ajtmh.23-0179

**Published:** 2023-08-14

**Authors:** Adam Abdullahi, James Frimpong, Mark T. K. Cheng, Sani H. Aliyu, Colette Smith, Alash’le Abimiku, Richard Odame Phillips, Michael Owusu, Ravindra K. Gupta

**Affiliations:** ^1^ Cambridge Institute of Therapeutic Immunology & Infectious Disease, Cambridge, United Kingdom;; ^2^ Department of Medicine, University of Cambridge, Cambridge, United Kingdom;; ^3^ Institute of Human Virology, Abuja, Nigeria;; ^4^ Kwame Nkrumah University of Science and Technology, Kumasi, Ghana;; ^5^ Kumasi Centre for Collaborative Research in Tropical Medicine, Kumasi, Ghana;; ^6^ Addenbrooke’s Hospital, Cambridge University Hospitals NHS Foundation Trust, Cambridge, United Kingdom;; ^7^ University College London, London, United Kingdom;; ^8^ Africa Health Research Institute, Durban, South Africa

## Abstract

Determination of previous SARS-COV-2 infection is hampered by the absence of a standardized test. The marker used to assess previous exposure is IgG antibody to the nucleocapsid (IgG anti-N), although it is known to wane quickly from peripheral blood. The accuracies of seven antibody tests (virus neutralization test, IgG anti-N, IgG anti-spike [anti-S], IgG anti–receptor binding domain [anti-RBD], IgG anti-N + anti-RBD, IgG anti-N + anti-S, and IgG anti-S + anti-RBD), either singly or in combination, were evaluated on 502 cryopreserved serum samples collected before the COVID-19 vaccination rollout in Kumasi, Ghana. The accuracy of each index test was measured using a composite reference standard based on a combination of neutralization test and IgG anti-N antibody tests. According to the composite reference, 262 participants were previously exposed; the most sensitive test was the virus neutralization test, with 95.4% sensitivity (95% CI: 93.6–97.3), followed by 79.0% for IgG anti-N + anti-S (95% CI: 76.3–83.3). The most specific tests were virus neutralization and IgG anti-N, both with 100% specificity. Viral neutralization and IgG anti-N + anti-S were the overall most accurate tests, with specificity/sensitivity of 100/95.2% and 79.0/92.1%, respectively. Our findings indicate that IgG anti-N alone is an inadequate marker of prior exposure to SARS COV-2 in this population. Virus neutralization assay appears to be the most accurate assay in discerning prior infection. A combination of IgG anti-N and IgG anti-S is also accurate and suited for assessment of SARS COV-2 exposure in low-resource settings.

## INTRODUCTION

The coronavirus disease 2019 (COVID-19) continues to affect the global community at an unprecedented rate. Although vaccines designed against SARS COV-2 have been the cornerstone of the global strategy against COVID-19, limited vaccine supply, poor vaccine uptake, and vaccine hesitancy has led to delays in achieving population-level immunity in selected populations,^1^^,^^2^ especially across the sub-Saharan African region. One study previously showed that almost half of healthcare workers and one-third of unvaccinated participants were previously infected with SARS COV-2, using anti-nucleocapsid (anti-N) seropositivity prior to national vaccine rollout in Nigeria and Ghana, respectively.^3^

Exposure to SARS COV-2 antigens driven by previous exposure shapes immunity to SARS COV-2 in individuals and at the population level.^4^ Anti-N and anti-spike (anti-S) markers appear after infection and are used to differentiate between immune responses elicited by infection or vaccination.^5^ The natural course of SARS COV-2 infection involves the appearance and persistence of anti-N and anti-S markers for several months post-infection.^6^^–^^9^ It has been observed that the anti-N antibody has a shorter half-life and persistence than anti-S antibodies,^7^ with the anti-N antibody peaking at around 30 days post-infection.^6^ Further, evidence from East Africa has shown that the anti-N antibody did not appear among one in four study participants within 1 month of a confirmed polymerase chain reaction (PCR) test.^10^

Recognizing unvaccinated individuals with previous infection is an important component of managing SARS COV-2 infection as it relates to contact tracing, isolation, and refining the appropriate intervention strategies for vaccination and case management, especially where PCR testing is not readily available and accessible at the population level. It should be noted that vaccination coverage is still patchy; for example, only 59.3% in Nigeria and 44% in Ghana of the eligible population are fully vaccinated.^11^ In this study, using cross-sectional samples collected prior to vaccination rollout in Kumasi, Ghana, in 2021, we characterized four markers of SARS COV-2, including serum virus neutralization titer, total IgG binding antibodies for anti-S, IgG anti-N, and IgG anti–receptor binding domain (anti-RBD), with the aim of evaluating the ideal marker or combination of markers of characterizing previous SARS COV-2 infection.

## MATERIALS AND METHODS

### Study design and participants.

Our study was a retrospective cross-sectional comparative analysis of markers of SARS COV-2 on bio-archived anonymized samples. The study cohort comprised 502 participants from the general population of the Kumasi area as previously described.^3^ These participants were earmarked to receive the SARS COV-2 ChadOx-1 (AstraZeneca) vaccine in early 2021. Study participants provided written informed consent, and blood samples were collected according to the protocols approved by the Committee of Human Research, Publication and Ethics of KNUST (CHRPE/AP/091/21).

### Binding antibody and viral neutralization antibody testing.

We measured binding IgG antibodies against SARS-COV-2 receptor-binding domain (RBD), total trimeric spike protein (S), and nucleocapsid protein (N) using the Luminex-based SARS-CoV-2-IgG assay as we previously detailed.^12^^,^^13^ We defined the cutoff of each antibody using an analysis of “true” positive (convalescent) and negative pre-pandemic samples as we previously described.^3^ In brief, positive binding antibodies were defined using a threshold of 1,896, 456, and 6,104 mean fluorescence intensity for IgG anti-S, anti-RBD, and anti-N IgG, respectively.^3^ For plasma-neutralizing antibody measurement, SARS-CoV-2 virus (pseudotyped virus [PV]) was prepared by transfecting HEK293T cells with Wu-1-614G wild type using p8.91 HIV-1 gag-pol expression vector.^14^ Virus neutralization was performed on Hela-ACE2 cells using SARS-CoV-2 spike PV-expressing luciferase. Briefly, plasma samples were heat inactivated at 54°C for 1 hour, serially diluted in duplicate, and incubated with PVs at 37°C for 1 hour prior to addition of Hela-ACE2 cells.^15^ The plasma dilution/virus mix was incubated for 48 hours in a 5% CO_2_ environment at 37°C, and luminescence was measured using the Bright-Glo Luciferase assay system (Promega). All neutralization assays were repeated in two independent experiments containing two technical replicates for each condition. Neutralization was calculated relative to virus-only controls as a mean neutralization with standard error of the mean. The half maximum inhibitory dose (ID_50_) was calculated in GraphPad Prism version 9.3.1; ID_50_ > 20 was considered positive. 293T cells (ATCC: CRL-3216) and HELA-ACE2 cells were a kind gift from Dr. James Voss, SCRIPPS.

### Definition of “composite gold standard” and analyses.

In the absence of a “gold standard” for evaluating previous SARS COV-2 infection, we defined composite gold standard as positivity to either IgG anti-N or viral neutralization (ID_50_ > 50). The sensitivity of each “index test” was evaluated as the proportion of positive result over the positive specimens using the composite reference standard, and the specificity of each index test was evaluated as the proportion of negative results over the negative specimens using the composite reference standard using the defined thresholds. Uncertainty was quantified using 95% CIs, and corresponding receiver operating characteristic (ROC) graphs were plotted for each of the index tests.

## RESULTS

The study population of 502 participants had a median age of 33 years (interquartile range [IQR]: 25–47), of which the majority participants were male (280/502; 56.0). No significant difference in age was observed between the two population groups in terms of positivity using the composite gold standard as strata for previous exposure (*P* = 0.32). Of 502 pre-vaccination participant samples, 262 (52.2%) were positive as per composite gold standard and used as the denominator for sensitivity analysis, and 240 (47.8%) were negative and used as the denominator for specificity analysis.

Using the composite standard as reference and evaluating the accuracy of IgG anti-N as the most accepted marker of previous exposure, we found high percentages of false negatives (132/262, 50.4%). The proportion of participants who were true positives using the different markers either singly or in combination is shown in Table 1. It is important to note that viral neutralization assay (250/262; 95.4%) and IgG anti-N + anti-S (226/262; 86.3%) had the highest proportions of true positives (Table 2). The overall accuracy of index tests is summarized in Table 2. The most sensitive tests were the neutralization test and the combination of IgG anti-N + anti-S, with sensitivity of 95.4% and 79.0%, respectively; the least sensitive test was IgG anti-N alone at 52.7% (Table 2).

**Table 1:** Test accuracy of seven index tests using different markers of SARS COV-2 infection either singly or in combination

Test	Positives, *n* (% of total sample)	True positive, sensitivity (%)	Negatives, *n* (% of total sample)	True negatives, specificity (%)
Virus neutralization	250 (49.8)	250 (95.4)	252 (50.2)	240 (100)
IgG anti-N	138 (27.5)	138 (52.7)	364 (72.5)	240 (100)
IgG anti-S (total)	218 (43.4)	199 (76.0)	284 (56.6)	221 (92.1)
IgG anti-RBD	195 (38.8)	181 (69.1)	307 (61.2)	226 (94.2)
IgG anti-N + anti-RBD	213 (42.4)	199 (76.0)	289 (57.6)	226 (94.2)
IgG anti-N + anti-S	226 (45.0)	207 (79.0)	276 (55.0)	221 (92.1)
IgG anti-RBD + anti-S	223 (44.4)	201 (76.7)	279 (55.6)	218 (91.0)

IgG anti-N = immunoglobulin against SARS COV-2 N protein; IgG anti-RBD = immunoglobulin against SARS COV-2 receptor binding domain protein; IgG anti-S = immunoglobulin against SARS COV-2 total spike protein. Total population (*N* = 502); true positive according to composite reference standard (*n* = 262); true negative according to composite reference standard (*n* = 240).

**Table 2:** Test accuracy of seven index tests using different markers of SARS COV-2 infection either singly or in combination showing AUC values, specificity, sensitivity, PPV, and NPV with 95% CI

Test	AUC (95% CI)	Specificity (95% CI)	Sensitivity (95% CI)	PPV (95% CI)	NPV (95% CI)
Virus neutralization	0.98 (0.96–0.99)	100 (98.5–100)	95.4 (93.6–97.3)	100 (98.5–100)	95.2 (93.4–97.1)
IgG anti-N	0.76 (0.73–0.79)	100 (98.5–100)	52.7 (48.3–57.0)	100 (98.5–100)	66.0 (62.0–70.0)
IgG anti-S (total)	0.84 (0.81–0.87)	92.1 (89.7–94.5)	76.0 (72.2–80.0)	91.3 (88.8–93.6)	77.8 (74.1–81.5)
IgG anti-RBD	0.82 (0.78–0.85)	94.2 (92.1–96.2)	69.1 (65.0–73.1)	92.8 (90.6–95.1)	73.6 (69.8–77.5)
IgG anti-N + anti-RBD	0.85 (0.82–0.88)	94.2 (92.1–96.2)	76.0 (72.2–79.7)	93.4 (91.3–95.6)	78.2 (74.6–81.8)
IgG anti-N + anti-S	0.86 (0.83–0.89)	92.1 (89.7–94.5)	79.0 (75.5–82.6)	91.6 (89.2—94.0)	80.0 (76.6–83.6)
IgG anti-RBD + anti-S	0.84 (0.80–0.87)	91.0 (88.3–93.4)	76.7 (73.0–80.4)	90.1 (87.5–92.7)	78.1 (74.5–56.6)

AUC = area under the curve; IgG anti-N = immunoglobulin against SARS COV-2 N protein; IgG anti-RBD= immunoglobulin against SARS COV-2 receptor binding domain protein; IgG anti-S = immunoglobulin against SARS COV-2 total spike protein; NPV = negative predictive value; PPV = positive predictive value. Total population, *N* = 502; positive according to composite reference standard, *n* = 262.

**Figure 1.**
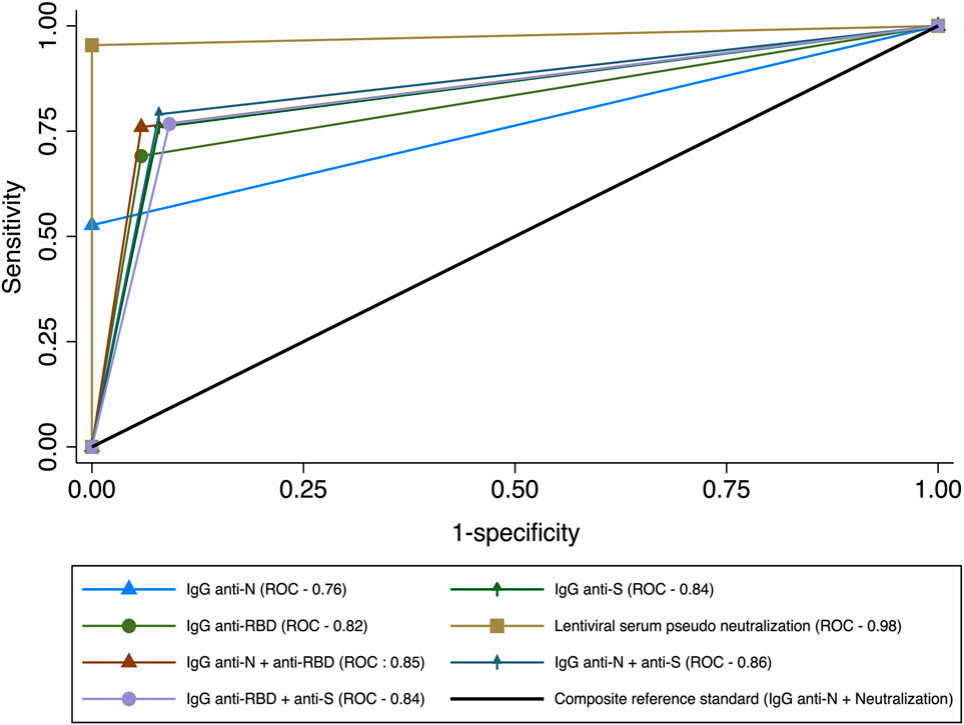
Receiver operating characteristic analyses showing the area under the curve across seven index tests used to evaluate the best markers of SARS COV-2 exposure. Positivity to either IgG anti-N or detectable virus neutralization was used as composite gold standard.

Regarding specificity, IgG anti-N and virus neutralization were the most specific tests (100%) according to our composite gold standard, followed by the combination of IgG anti-N + IgG anti-RBD and IgG anti-N at 94%. The other index tests were also highly specific at > 90%. The virus neutralization test and IgG anti-N had the highest positive predictive values (PPVs) at 100%, and the serum neutralization test (95.2%) and IgG anti-N + anti-S (80.0%) had the highest negative predictive values (NPVs) (the PPV and NPV are dependent on the population prevalence of prior COVID-19 infection). Comparing the ROC curves for each index test’s ability to discriminate previous exposure (Figure 1), virus neutralization test still had the highest area under the curve (AUC: 0.98), reflective of excellent discriminatory ability to exposure to SARS COV-2. Other index tests had acceptable AUC values above 0.82, with IgG anti-N antibodies giving an AUC value of 0.76 (Figure 1).

## DISCUSSION

Accurate characterization of previous exposure to SARS COV-2 and other related respiratory pathogen exposure is a critical component of pandemic management, including accurate evaluation of population-level seroprevalence to inform public health interventions/policy^16^ and vaccination strategies, including possible fractional dosing vaccination approaches.^17^^,^^18^ We show definitive evidence from a West African population of the indiscriminatory capacity of the IgG anti-N to accurately characterize previous exposure to SARS COV-2, which is likely to lead to an underestimation of seroprevalence and previous exposure.

In this present study, we showed the most accurate test based on sensitivity and specificity was PV neutralization, with 95% sensitivity and 100% specificity. This was consistent with the PPV and NPV of 100% and 95%, respectively. Accurate detection of different markers of SARS COV-2 for characterization of previous exposure has its advantages and drawbacks. Although we used a highly sensitive Luminex approach to measure binding antibodies, we believe that serological markers measured directly at the bench using ELISA should produce similar results. These tests have a quick turnaround time and accuracy (sensitivity and specificity), although the inability to discern between coronaviruses responses resulting from cross-reactivity due to viral sequence homology^19^^,^^20^ is a particular concern.

As the SARS COV-2 pandemic continues, novel variants of concern with mutations enhancing transmissibility, replication, and immunity evasion capacity continue to emerge as observed in the Delta^21^^–^^25^ and Omicron variants,^15^ deriving from chronic infections.^26^ These novel variants may be associated with a difference in the antibody kinetics of markers, such as the anti-N observed with neutralization responses. Likewise, as vaccination coverage expands globally, the emergence of novel variants that are likely to lead to breakthrough infection in already vaccinated or previously infected individuals will become increasingly common,^27^^,^^28^ which further affects antibody kinetics. It is noteworthy that during the early phase of vaccination scale-up, there were limited reported cases of breakthrough infections in high-income settings;^29^^,^^30^ however, this changed with the arrival of the immunity-evasive Delta variant.^21^^,^^25^^,^^31^^,^^32^ Similarly, in our previous analysis from Nigeria, we observed a breakthrough infection rate of 16% following two doses of the Chad-Ox1 vaccine in a healthcare worker cohort^3^ during the Delta variant wave of 2021 and relatively similar rates were observed in Uganda^33^.

Data on the longitudinal trajectories of SARS COV-2 IgG antibodies in western cohorts are conflicting. Two reports showed sustained responses to IgG spike and nucleocapsid up to 125 days after exposure,^34^^,^^35^ whereas other reports reported declines in antibody levels over the same time period,^36^^–^^38^ with limited antibody data. In our previous analysis in a West African population comprising 140 and 527 participants from Nigeria and Ghana and using IgG anti-N, we reported seroprevalence rates of 44% and 28%, respectively, which increased to 59% and 39% when IgG anti-RBD was used as an additional marker of previous infection due to its specificity for the SARS COV-2 epitope.^3^ Our data from Ghana are consistent with evidence from Lagos in participants from the general population prior to vaccination rollout in early 2021, which showed underestimation of previous infection when SARS-CoV-2 IgG anti S + anti-N or IgG anti–S-only responses were measured by T-cell interferon-γ assay for use as a diagnostic biomarker for previous exposure.^39^ T cell responses have also been observed in antibody negative participants in Kenya.^40^ This indicates that anti-N is not an ideal independent marker and that additional markers are required for accurate discernment of previous exposure, although time since exposure event is likely to play a role because the antibody response wanes with time.

Our study size is robust with over 500 participants, although demographic data were limited to sex and age. Previous evidence has shown age-related predictors (likely driven by immune senescence) of waning humoral response to vaccination and, in principle, humoral responses triggered by previous SARS COV-2.^40^^–^^43^ We found no difference between the ages of participants classified positive or negative by composite gold standard (i.e., median age of 34 [IQR: 26–47] and 33 [IQR: 25–47], respectively; *P* = 0.32); this is reassuring and suggests that study participants likely have robust immune systems, with similar waning rate between groups.

This study was subject to limitations. It was advantageous to have access to a large cohort of samples collected pre-vaccination within a West African setting, which is understudied. We note the collection of limited study demographic data and the lack of administered questionnaires, which could have included self-reported measures of potentially previous exposure. Finally, our use of a Luminex high-performance assay for IgG antibody measurement should be considered more than a simplified approach especially in the absence of a comparative analysis with standard on the bench ELISA kits of comparable accuracy.

We conclude that IgG anti-N alone is an inadequate marker of prior exposure to SARS COV-2 in an unvaccinated population with an underestimation of previous exposure by almost 50%. In our hands, virus neutralization assay was the most accurate assay in discerning prior infection in unvaccinated populations. In resource-limited settings where virus neutralization assay is not easily available, a combination of IgG anti-N and IgG anti-S is potentially a suitable alternative.
